# Ultrasensitive, light-induced reversible multidimensional biosensing using THz metasurfaces hybridized with patterned graphene and perovskite

**DOI:** 10.1515/nanoph-2021-0816

**Published:** 2022-02-11

**Authors:** Haiyun Yao, Zhaoqing Sun, Xin Yan, Maosheng Yang, Lanju Liang, Guohong Ma, Ju Gao, Tenten Li, Xiaoxian Song, Haiting Zhang, Qili Yang, Xiaofei Hu, Ziqun Wang, Zhenhua Li, Jianquan Yao

**Affiliations:** School of Opto-electronic Engineering, Zaozhuang University, Zaozhuang, 277160, China; Faculty of Materials and Manufacturing, Beijing University of Technology, Beijing, 100124, China; School of Information Science and Engineering, Zaozhuang University, Zaozhuang, 277160, China; School of Electrical and Optoelectronic Engineering, West Anhui University, Lu’an, 237000, China; Department of Physics, Instrumental Analysis & Research Center, Shanghai University, Shanghai, 200444, China; College of Precision Instruments and Opto-electronics Engineering *,* Tianjin University *,* Tianjin, 300072, China; Institute of Micro-nano Optoelectronics and Terahertz Technology, Jiangsu University, Zhenjiang, 212013, China

**Keywords:** biosensor, EIT, reversible, THz metasurfaces, whey protein

## Abstract

Biosensors based on terahertz (THz) metasurfaces have recently attracted widespread attention. However, few have been reported so far because it is a challenge to achieve ultrasensitive multidimensional detection in the THz spectrum. Here, we propose a novel THz biosensor that consists of a metasurfaces and a metal oxide semiconductor-like structure (MOSLS), which is based on patterned graphene–polyimide–perovskite. We varied the photoconductivity of the MOSLS via the electrostatic doping effect. The biosensor could detect whey protein down to a concentration limit of 6.25 ng/mL. Significant responses in frequency, phase, and transmission amplitude were all detected for different protein concentrations. The transmission value difference, frequency shift, and phase difference increased with the concentration of whey protein, clearly demonstrating multidimensional biosensing. Moreover, by applying lasers with different wavelengths, we have realized reversible biosensing in THz region for the first time. These results are very promising for applications of THz metasurfaces in the field of biosensing.

## 1 Introduction

Metasurfaces have attracted extensive attention as a novel way to manipulate electromagnetic waves for diverse applications, including as modulators [1], [2], [3], [4], [5], absorbers [6, 7], and biosensors [8], [9], [10], [11], [12], [13], [14]. The primary mechanism is that the resonant modes of metasurfaces are susceptible to changes in their microenvironment. In particular, the absorbance linewidth of their electromagnetically induced transparency (EIT) is limited only by Drude damping. The EIT-like features of metasurfaces make them ideal for ultrasensitive biosensors [9, 13]. Because the characteristic vibrational modes of numerous macromolecules like proteins, DNA, and viruses are in the THz spectrum, THz metasurfaces are particularly desirable [9, 15].

Nevertheless, the development of metasurface-based THz biosensing systems is still in its initial stages [16], [17], [18], [19], [20]. Previous works focused mainly on change transmission amplitude or shift resonance frequency in metasurfaces as the sole sensing index. However, the poor sensitivity of these materials must be improved to be useful as an actual application index. Phase difference is rarely reported as a sensing index. New materials and mechanisms combined with THz metasurfaces are needed to achieve ultrasensitive, multidimensional (frequency, amplitude, phase) detection of biological macromolecules.

Recently, graphene has attracted much interest because it presents several unique advantages: large specific surface area, tunable optoelectronic properties, and good biocompatibility [16, 19, 21], [22], [23]. Patterned graphene with well-defined architecture on the micrometer to submicrometer scale has become increasingly important because of its low-loss plasma characteristics [24], [25], [26]. The combination of patterned graphene and THz metasurfaces shows great potential for ultrasensitive biosensing [27]. However, simply combining graphene and metasurfaces is no longer sufficient to meet the needs of this field because it is not easy to achieve phase modulation with such materials.

At present, most research on phase-change materials focuses on metal halide perovskites [28, 29] (ABX_3,_ where A = methylamine(MA), B = Pb or Sn; and X = Cl, Br, or I). They have outstanding optoelectronic properties [30], such as excellent charge-carrier mobilities, bandgap tunability, and high photoluminescence quantum yield. Effectively combining the patterned graphene layer with a second optical material, especially ABX_3_, is a new possibility for the design of THz biosensors. Such composite materials can not only enable multidimensional sensing but also achieve ultrasensitive detection.

**Figure 1:**
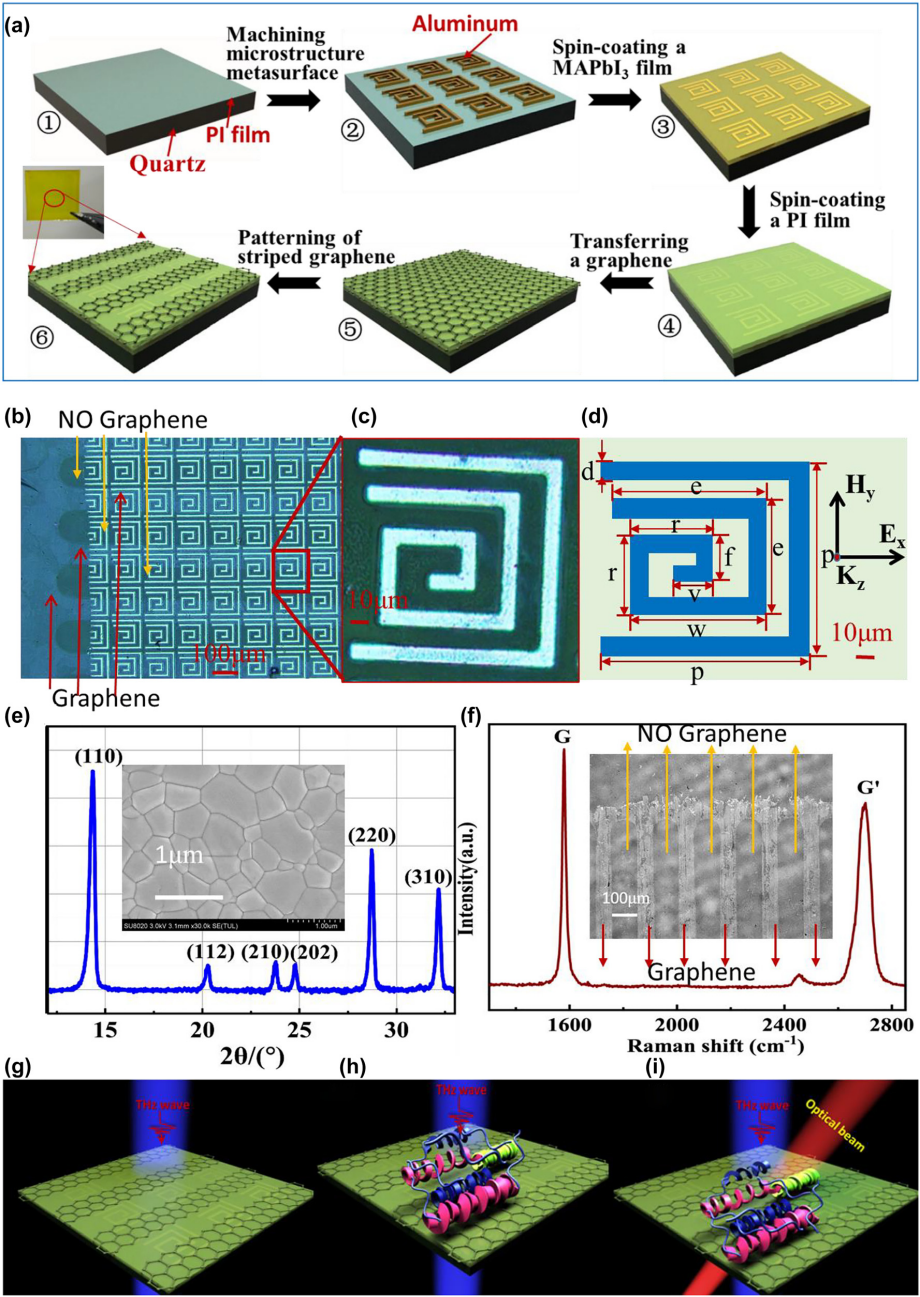
Fabrication and characterization of the proposed PGPP@MS biosensor. (a) The fabrication process. ① A PI film was spin-coated on top of a quartz substrate. ② The metasurface, an asymmetric Al microstructure, was machined atop the PI film. ③ A metal halide perovskite (MAPbI_3_) film was spin-coated on the metasurface, covering the microstructure. ④ The PI film was spin-coated onto the MAPbI_3_ film. ⑤ A graphene layer was transferred onto the PI film. ⑥ The graphene was patterned into stripes. (b) Photomicrograph of a small region of the PGPP@MS biosensor and the patterned graphene (dark blue regions indicated by red arrows). (c) Photomicrograph of a unit cell of the microstructure. (d) Schematic of the unit cell, which consists of an external U shape and an internal asymmetric spiral. The geometric parameters are, for the U shape: *p* = 135 μm and *d* = 13 μm; and for the spiral structure: *e* = 90 μm, *w* = 103 μm, *r* = 63 μm, *f* = 39 μm, and *v* = 31 μm. The periodicity is 150 µm. (e) X-ray diffraction (XRD) pattern for the MAPbI_3_ film. Inset: Scanning electron microscopy (SEM) image of the MAPbI_3_ film. (f) Raman spectrum of graphene. Inset: Photomicrograph of striped graphene. (g)–(i) Schematic illustration of the proposed biosensor. THz beams penetrate the device without whey protein at normal incidence (g). Different concentrations of protein can be deposed on the surface (h), and an external optical pump can be applied after measurement to allow reversible sensing (i).

Here, we propose a novel biosensor that integrates metal oxide semiconductor-like structures (MOSLS) based on patterned graphene–polyimide (PI)–perovskite (MAPbI_3_) with EIT-like metasurfaces (later the biosensor will be labeled by PGPP@MS) to manipulate THz waves and realize ultra-sensitive, multidimensional sensing, as illustrated in Figure 1 [31, 32]. The PI film ensures the stability of MAPbI_3_ under ambient conditions and provides stable support for the graphene. The proposed EIT-like metasurfaces enable the generation of two polarization-independent transparent windows at 0.63 THz and 1.16 THz, and may also enhance sensitivity.

As a proof of concept, whey protein is adhered to the patterned graphene of the proposed PGPP@MS biosensor and the THz transmission spectra are measured. By varying the photoconductivity of the MOSLS via the electrostatic doping (ED) effect [33, 34], frequency, phase, and amplitude responses for different concentrations of whey protein (CWP) are obtained. Our PGPP@MS biosensor can detect CWPs as low as 6.25 ng/mL.

Furthermore, the transmission value difference of (Δ*V*_T_), frequency shift (Δ*ƒ*), and phase difference (Δ*P*) all increase with CWP between 6.25 ng/mL and 316 μg/mL, successfully realizing multidimensional biosensing. In addition, we have realized reversible biosensing in the THz region by illuminating the biosensor with lasers at different wavelengths. These results show great promise for applications of THz metasurfaces in the field of biosensing.

## 2 Materials and methods

### 2.1 Design of the PGPP@MS biosensor

**Figure 2:**
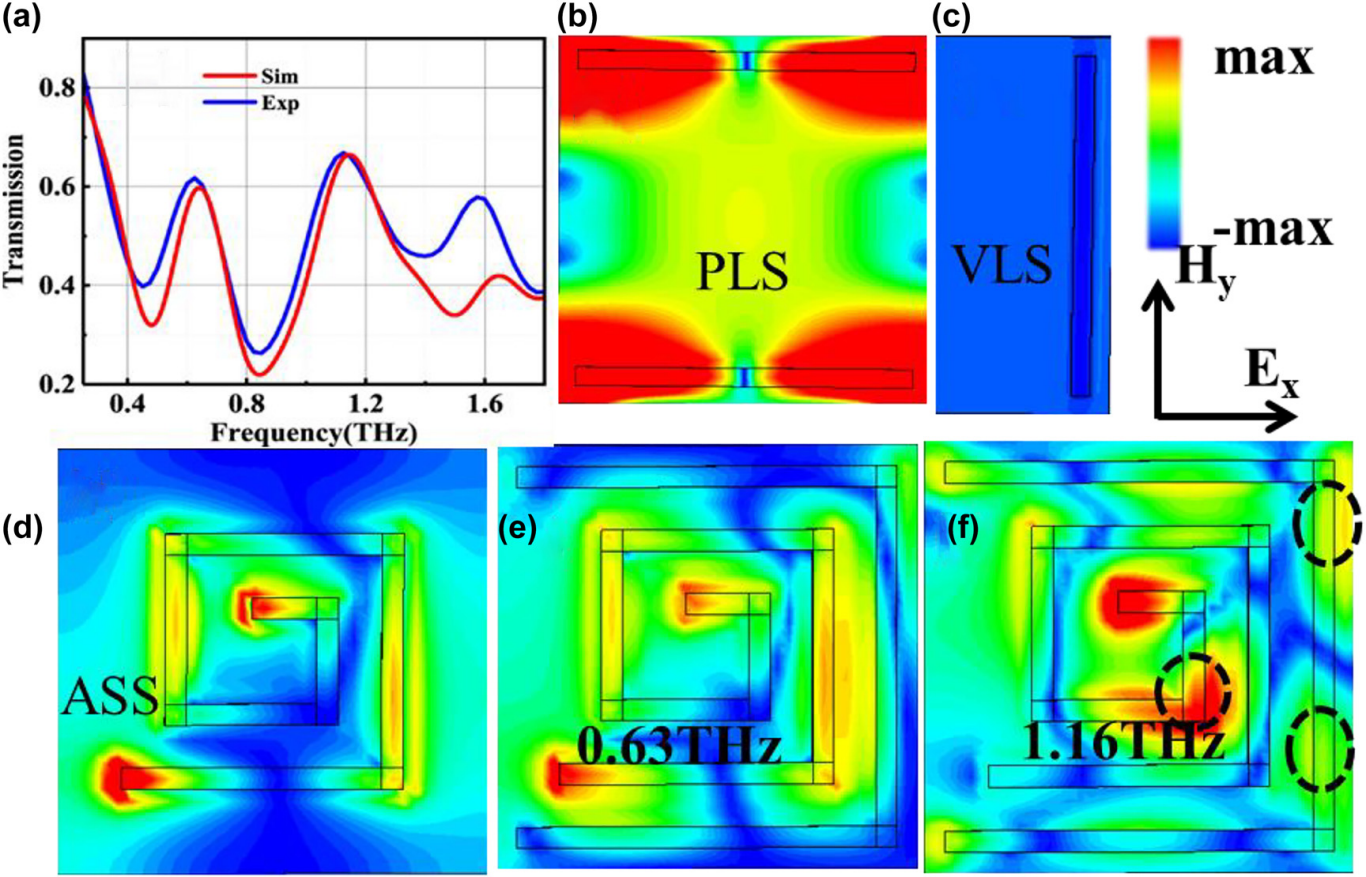
Simulated electric field intensity distribution of the metasurfaces: (a) experimental (blue line) and simulated (red line) transmission spectra of the PGPP@MS biosensor sample. (b)–(d) Simulated electric field intensity distributions at 0.63 THz of the PLS (b), VLS (c), and ASS (d) arrays. (e) Simulated electric field intensity distribution of the EIT mode at 0.63 THz. (f) Simulated electric field intensity distribution of the EIT mode at 1.16 THz. The dashed circles highlight field intensity that is indicative of dark mode excitation. The entire incident light is *x*-polarized.

A schematic layout and all geometric parameters of the proposed PGPP@MS biosensor are depicted in Figure 1. Figure 1(a) shows an overview of the fabrication process for the PGPP@MS biosensor, which begins with a 5 μm-thick PI film spin-coated on a 300 μm-thick quartz substrate (1.0 cm × 1.0 cm) (Figure 1(a), ①). An array of 0.2 μm-thick aluminum microstructure units is fabricated atop the PI layer via standard photolithography methods (Figure 1(a), ②). The dimensions of the unit cell are shown in the photomicrographs in Figure 1(b)–(d). It consists of external U-shaped (parallel long sides (PLS) and vertical long side (VLS)) and internal asymmetric spiral structures (ASS).

**Figure 3:**
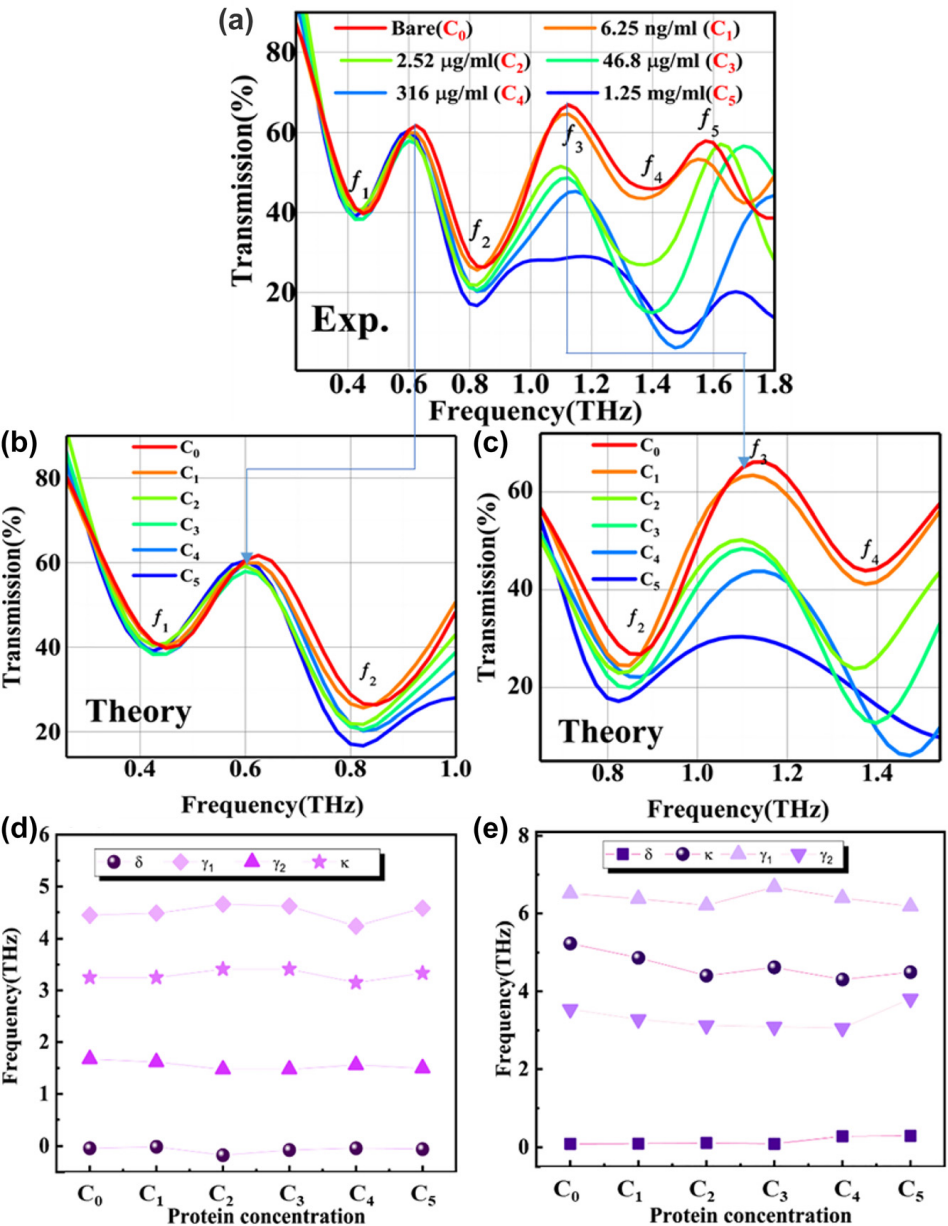
Experimentally measured and theoretically fitted for transmission spectra of the PGPP@MS biosensor at different CWP. (a) Experimentally measured THz transmission spectra of the PGPP@MS biosensor at different CWP. C_0_ = 0 ng/mL, C_1_ = 6.25 ng/mL, C_2_ = 2.52 μg/mL, C_3_ = 46.8 μg/mL, C_4_ = 316 μg/mL, and C_5_ = 1.25 mg/mL). Peaks *f*_1_–*f*_5_ are indicated to illustrate the sensing effect. (b) and (c) The transmission curves obtained by theoretically fitted at each CWP for two different EIT-like frequency bands, 0.25–1.0 THz (b) and 0.62–1.58 THz (c). (d) The CWP dependence of the oscillator parameters *γ*_1_, *γ*_2_, *δ*, and *κ*, extracted by fitting the transmission curves in (b) with Eq. (2). (e) As in (d) for the transmission curves in (c).

In the next step, a 0.25 μm-thick MAPbI_3_ film is spin-coated onto the metasurfaces, covering the microstructures (Figure 1(a), ③). This film shows good compactness and uniform morphology, with large grain sizes and no pinholes, as shown by the top-view SEM image in the inset of Figure 1(e). Figure 1(e) also shows the x-ray diffraction (XRD) pattern of the perovskite film. Diffraction peaks are located at 14.3°, 20.3°, 23.8°, 24.7°, 28.7°, and 32.1°, corresponding to the (110), (112), (210), (202), (220), and (310) crystal planes, respectively. No characteristic peaks associated with PbI_2_ or other redundant phases were observed.

**Figure 4:**
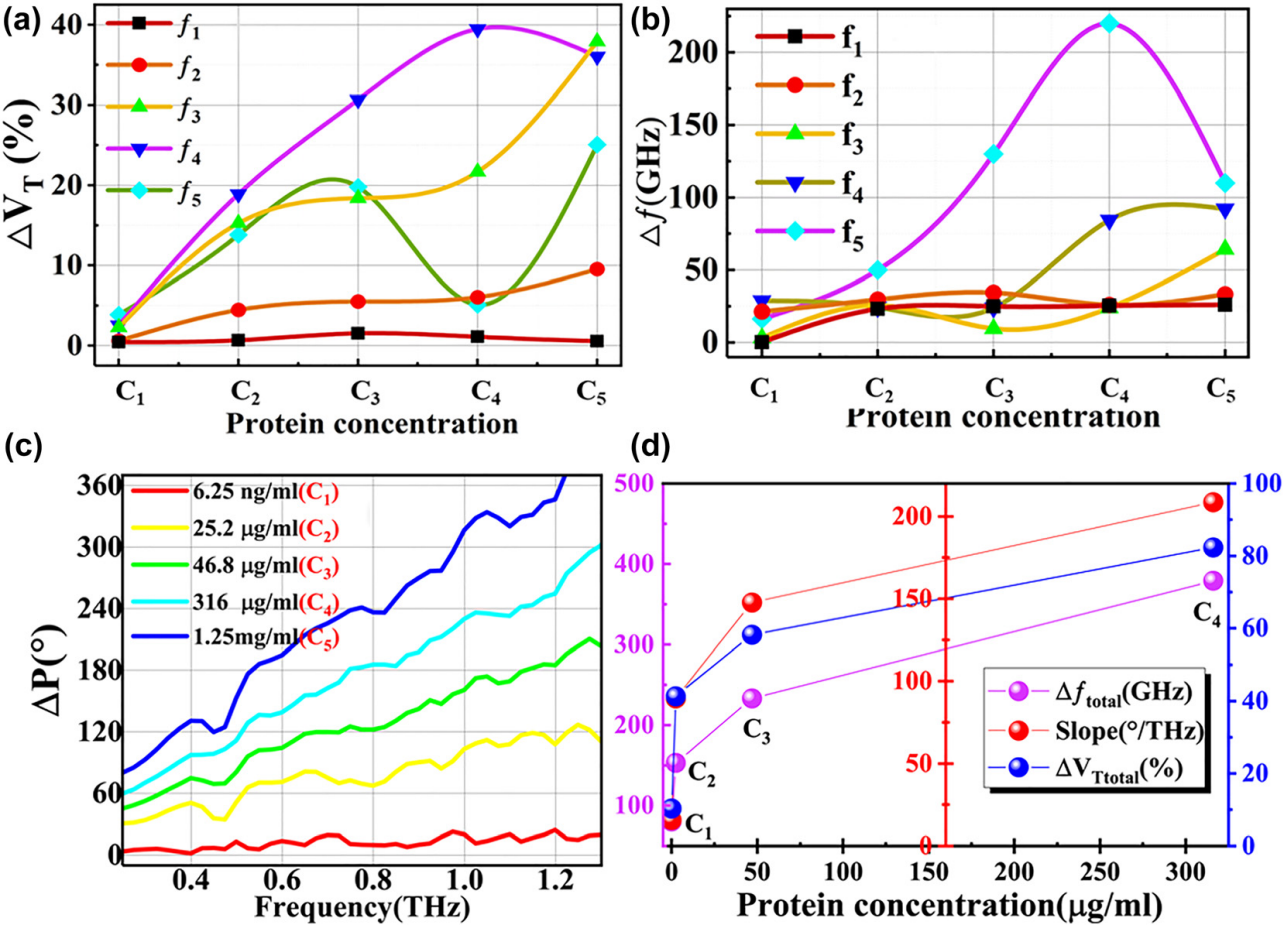
Quantitative analysis of multi-dimensional sensing performance. (a) and (b) The transmission value difference Δ*V*_T_ (a) and frequency shift Δƒ (b) between the bare sensor and each CWP between C_1_ and C_5_ at frequencies *f*_1_–*f*_5_. (c) Phase difference Δ*P* between the bare sensor and each CWP tested. (d) Summary of the CWP dependence of the physical properties in (a)–(c). Blue axis and markers: The sum of transmission value difference Δ*V*_Ttotal_ for all frequency points. Purple axis and markers: total frequency shift Δ*ƒ*_total_. Red axis and markers: fitted slopes of the phase difference, Δ*P*.

**Figure 5:**
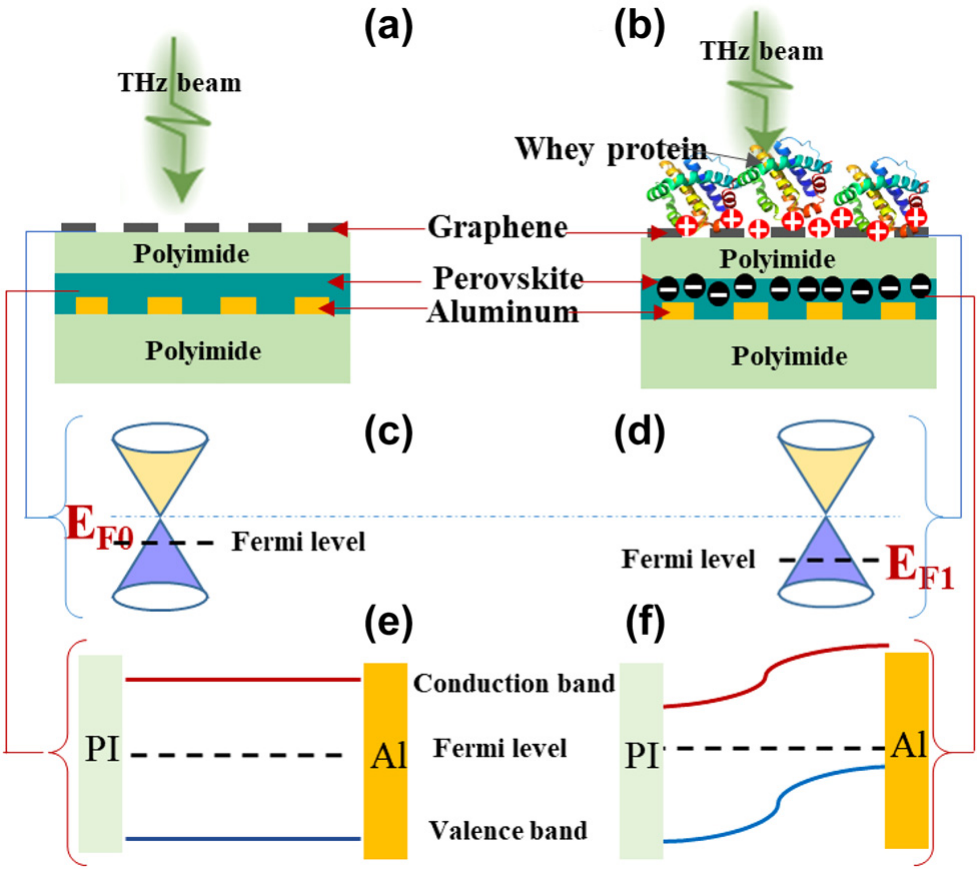
Schematic of the proposed sensing mechanism of the PGPP@MS biosensor. (a) and (b) Schematic diagram of the balanced state and charge distribution in PGPP@MS without (a) and with (b) whey protein. (c) and (d) Schematic diagrams of the Fermi level of graphene for PGPP@MS without (c) and with (d) whey protein. (e) and (f) Energy band of the perovskite layer for PGPP@MS without (e) and with (f) whey protein.

**Figure 6:**
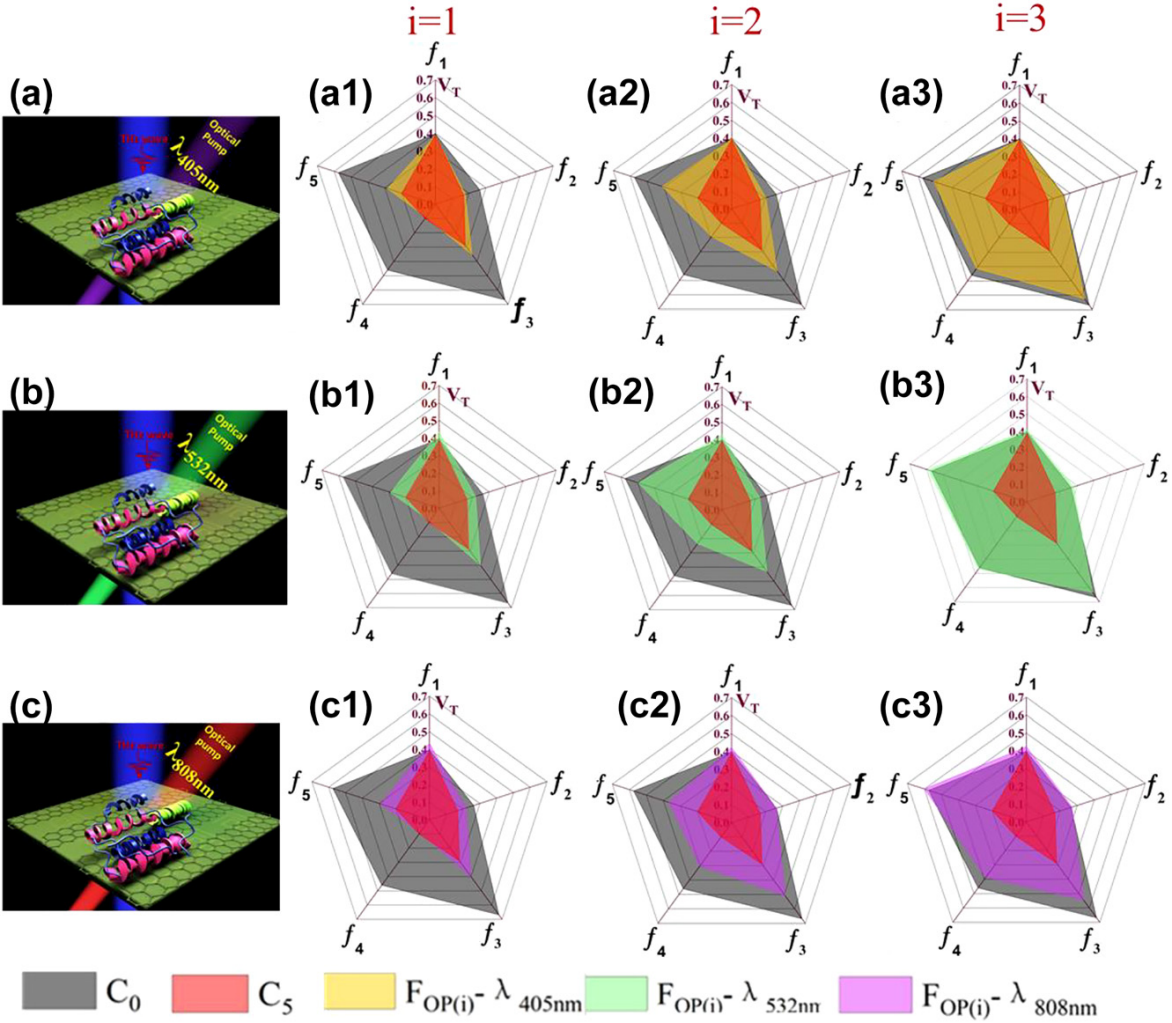
Description of reversible sensing. (a)–(c) Schematic diagrams of the PGPP@MS biosensor under THz beams and optical pumping at 405 nm (a), 532 nm (b), and 808 nm (c). (a1)–(a3) Pentagon radar maps of the transmission value (*V*_T_) at frequency peaks *f*_1_–*f*_5_ under different optical fluxes, *F*_op(i)_, at 405 nm. Every vertex in the radar maps represents *V*_T_ at a different frequency peak. Gray shapes represent *V*_T_ for the bare sensor, and red represents *V*_T_ when the protein concentration is C_5_. *V*_T_ after 405 nm illumination is shown in yellow. *i* = 1: *F*_op(1)_–λ_405 nm_ = 1.1 mW/cm^2^. *i* = 2: *F*_op(2)_–λ_405 nm_ = 11.5 mW/cm^2^. *i* = 3: *F*_op(3)_–*λ*_405 nm_ = 103.5 mW/cm^2^. (b1)–(b3) Radar maps for *V*_T_ following 532 nm illumination (shown in green; other colors are as in (a)). *i* = 1: *F*_op(1)_–*λ*_532 nm_ = 1.9 mW/cm^2^. *i* = 2: *F*_op(2)_–*λ*_532 nm_ = 42.4 mW/cm^2^. *i* = 3: *F*_op(3)_–*λ*_532 nm_ = 162.7 mW/cm^2^. (c1)–(c3) Radar maps for *V*_T_ following 808 nm illumination (shown in purple; other colors are as in (a)). *i* = 1: *F*_op(1)_–λ_808 nm_ = 2.2 mW/cm^2^. *i* = 2: *F*_op(2)_–*λ*_808nm_ = 28.3 mW/cm^2^. *i* = 3: *F*_op(3)_–*λ*_808 nm_ = 132.6 mW/cm^2^.

Next, a PI film was spin-coated atop the MAPbI_3_ layer (Figure 1(a), ④), which protects and isolates the MAPbI_3_. A 1.0 cm × 1.0 cm graphene layer was then transferred onto the top surface of the PI film (Figure 1(a), ⑤). Finally, for patterning of the graphene, zinc was sputtered onto the uppermost layer of graphene in 5 nm-thick stripes to remove a carbon layer selectively from those areas (Figure 1(a), ⑥). The device was placed in dilute HCl solution (about 0.05 M) for 1 min to dissolve the zinc. The device was then rinsed with water and dried in air. This series of steps removes one layer of carbon, and the procedure can be repeated to remove additional carbon layers. Ultimately, a graphene layer with a striped pattern is obtained. A photomicrograph of the striped graphene is shown in Figure 1(b) and as an inset to Figure 1(f).

Raman spectroscopy was performed at 514 nm excitation to confirm the quality of the graphene (Figure 1(f)). The ratio between the G peak (∼1578 cm^−1^) and the G′ peak (∼2699 cm^−1^) intensities is about 1.28. The full width at half maximum of the G′ peak is 54 cm^−1^. These features indicate that the graphene is of good quality [35]. The conductivity of graphene is about ∼1.7 × 10^6^ S/m (square resistance ∼600 Ω).

The resonant features of the PGPP@MS sample were experimentally characterized with an 8f confocal terahertz time-domain spectroscopy system (see detail in Figures S1 and S2). A 405 nm/532 nm/808 nm laser with a 3 mm spot diameter was used as an optical pump (Figure 1(i)).

Whey protein was chosen as a probe analyte to verify the performance of the PGPP@MS biosensor (Figure 1(h) and (i)). We prepared five different CWP in suspension, C_1_ = 6.25 ng/mL, C_2_ = 2.52 μg/mL, C_3_ = 46.8 μg/mL, C_4_ = 316 μg/mL, and C_5_ = 1.25 mg/mL. All the experimental data presented in this work are averaged after 3 tests.

### 2.2 Simulation of the PGPP@MS biosensor

We performed full-wave simulations with a frequency-domain solver based on finite-differential time-domain methods using CST Microwave Studio. The electric and magnetic boundary conditions were along the *x* and *y* directions (Figure 1(d)), respectively. The polarization direction of incident THz radiation was set along the *z* direction. In the simulation model, the quartz substrate was treated as a lossless dielectric with dielectric permittivity *ε* = 3.84 [36]. We used the classical Drude model to describe the conductivity of the graphene [37, 38]. The thickness of the graphene was set at 1 nm. The refractive index of polyimide was 3.1. The tangent loss was 0.05. The transmission *T*(*ω*) was defined as *T*(*ω*) = |*E*_s_(*ω*)/*E*_r_(*ω*)|^2^, where *E*_s_(*ω*) and *E*_r_(ω) are the THz electric field amplitudes of the sample and reference, respectively, after fast Fourier transformation of the THz pulses.

## 3 Results and discussion

### 3.1 Properties of the PGPP@MS biosensor

To investigate the underlying resonant mechanism of the PGPP@MS metasurfaces, the transmission spectra of the PGPP@MS biosensor were both simulated and measured, as shown in Figure 2(a). The experimental and simulated results agree well except for a slight difference in intensity at some resonance frequencies. This deviation may have been caused by fabrication errors or differences between the simulation conditions and the actual conditions. It is also interesting to note that a transmission peak with 60% efficiency appears at around 0.63 THz and 1.16 THz, as shown in Figure 2(a). This weak transparency phenomenon reasonably suggests that there may be an EIT-like mode-coupling effect in our metasurfaces [13, 39].

To further understand the physical mechanism of the EIT-like resonances, the electric field intensities of the PLS array, VLS array, and ASS array were simulated and shown in Figure 2(b)–(d), respectively. The field is mainly distributed at the four ends of the long sides of the PLS (Figure 2(b)) and localized around the starting and ending points of the ASS (Figure 2(d)). The VLS has no electric field distribution (Figure 2(c)). Therefore, the PLS acts as the bright mode, the VLS acts as the dark mode, and the ASS acts as the quasi-dark mode, simultaneously [40]. Combining the fields of the three coupled resonators results in two transmission windows around 0.63 THz and 1.16 THz, shown in Figure 2(a).

Figure 2(e) clearly shows that the electric field intensity in the PLS is entirely suppressed at these two frequencies as a result of their destructive interference. At 0.63 THz, the field intensity is mainly localized around the starting and ending points of the ASS, indicating the excitation of the quasi-dark mode. However, at 1.16 THz, the electric field intensity is mainly concentrated around the starting point, the right corner of the ASS, and two regions on the right side (black dotted circles in Figure 2(f)), suggesting that the dark mode is now excited. Therefore, our metamaterial can produce these two EIT-like modes under *x*-polarized incident light as a result of the destructive interference of the PLS (the bright mode), the ASS (the quasi-dark mode), and the VLS (the dark mode).

### 3.2 Sensing performance of the PGPP@MS biosensor

To investigate the sensing performance of the PGPP@MS biosensor, whey protein at various concentrations in aqueous solution was dropped onto the surface of the device, and the experimental test was performed after the water evaporation was completed.

Concentrations of 0 ng/mL (C_0_), 6.25 ng/mL (C_1_), 2.52 μg/mL (C_2_), 46.8 μg/mL (C_3_), 316 μg/mL (C_4_), and 1.25 mg/mL (C_5_) were tested. The transmission spectra of the PGPP@MS biosensor covered by different CWP are shown in Figure 3(a). The overall trend of the results is that transmission value decreases with increasing CWP.

Based on the THz transmission spectra of the PGPP@MS biosensor, we chose to examine shifts in the transmission spectrum at five frequencies, *f*_1_ = 0.42 THz, *f*_2_ = 0.81 THz, *f*_3_ = 1.16 THz, *f*_4_ = 1.39 THz, and *f*_5_ = 1.58 THz. Variations in both the frequency and the magnitude of the EIT peak occurred in the transmission spectrum as a function of CWP. At frequency points ƒ_3_, ƒ_4_, and ƒ_5_, the changes in transmission and peak frequency shifts were relatively large. In particular, ultra-sensitive detection of a very low CWP of 6.25 ng/mL was possible. We compare the sensitivity and sensing type of previous work mainly based on the interaction of external molecules with graphene (see Table 1). WP molecules have no benzene ring like structures and without *π*-electrons. Therefore, it cannot form *π*–*π* stacking with graphene. First, we compared the case without *π*–*π* stacking, and from Table 1, it can find that the sensitivity of our designed sensor is two orders of magnitude higher than the other two sensors. And, our biosensor realizes three-dimensional sensing. Generally, if an external molecule has a benzene ring like structure with *π*-electrons, it strongly interacts with the *π*-electrons of graphene through *π*–*π* stacking [19]. Therefore, the graphene-based sensor show higher sensitivity to the external molecules containing *π*-electrons [19]. However, the sensitivity of our designed sensor to detect WP molecules without *π*-electrons is higher than that of detecting CMM which form *π*–*π* stacking with graphene in previous work. In summary, our designed sensor has higher sensitive sensing performance.

**Table 1:** Comparison with previous works based on THz sensors.

Material	Analytes	LOD	Sensing type	IMG	[Reference]
**MOSLS + MS (EIT)**	**WP**	**6.25 ng/ml**	**Amplitude+**	No *π*–*π* stacking	**This work**
			**Frequency + Phase**		
MS (EIT)	MDK	0.5** **ug/ml	Amplitude + frequency	No *π*–*π* stacking	[41]
Graphene + MS	Fructose	100 ng/ml	Amplitude	No *π*–*π* stacking	[42]
Graphene + PI	CMM	130 ng/ml	Amplitude	*π*–*π* stacking	[19]
Graphene + MS	CMM	20 ng/ml	Amplitude	*π*–*π* stacking	[42]

LOD, limit of detection; IMG, the interaction between external molecules and graphene; WP, whey protein. MS, metasurface; EIT, electromagnetically induced transparency; PI, polyimide; MDK, Midkine; CMM, chlorpyrifos methyl molecules.

The effect of the analyte on the behavior of the PGPP@MS biosensor can be explained by the coupled harmonic oscillator model, which describes the near-field coupling between bright- and dark-mode resonators. The coupled differential equations can analytically describe the interference [43]:

$${\ddot{x}}_{1}+\gamma_{1}{\dot{x}}_{1}+\omega_{0}^{2}x_{1}+\kappa x_{2}=E\text{ ,}$$

(1) $${\ddot{x}}_{2}+\gamma_{2}{\dot{x}}_{2}+{\left(\omega_{0}+\delta \right)}^{2}x_{2}+\kappa x_{1}=0\text{ .}$$

Here, *x*_1_, *x*_2_, *γ*_1_, and *γ*_2_ are the resonant amplitudes and losses of the bright and dark modes, respectively. *ω*_0_ is the resonance frequency of the bright mode oscillator (*γ*_2_ « *γ*_1_ « *ω*_0_), *δ* denotes the detuning of the resonant frequency of the dark mode oscillator from the bright mode (*δ* « *γ*_1_), and *κ* is the coupling coefficient between the two oscillators. By solving Eq. (1) with the approximation *ω* − *ω*_0_ « *ω*_0_, the susceptibility *χ* is obtained:

(2) $$\chi =\chi_{r}+i\chi_{i}\propto \frac{\left(\omega +\omega_{0}-\delta \right)+\frac{i\gamma_{2}}{2}}{\left(\omega -\omega_{0}+\frac{i\gamma_{1}}{2}\right)\left(\omega -\omega_{0}-\delta +\frac{i\gamma_{2}}{2}\right)-\frac{\kappa^{2}}{4}}$$

where *χ*_r_ is the real part of the susceptibility, and *χ*_i_ is the imaginary part, which is proportional to the energy losses. The transmission *T* can be obtained through *T* = 1 − *gχ*_i_ [44], where *g* is the geometric parameter that indicates the coupling strength of the bright mode with the incident electric field *E*.

We defined two frequency bands, from 0.25 THz to 1 THz and from 0.62 THz to 1.58 THz, as two distinct regions of EIT-like effects. The measured transmission spectra of the PGPP@MS biosensor in these regions were theoretically fitted with Eq. (2), as shown in Figure 3(b) and (c). The fitting results show reasonable consistency with the experiments.

The fitting parameters as a function of the CWP are shown in Figure 3(d) and (e) for the lower- and higher-frequency bands, respectively. It can be observed from Figure 3(d) that *δ*, *κ*, and *γ*_2_ are almost constant in the low-frequency band, whereas *γ*_1_ changes slightly with increasing CWP. The radiative damping of the bright mode resonator, *γ*_1_, decreases to 4.1 THz at C_4_, compared with 4.5 THz for a bare sensor.

In the high-frequency band (Figure 3(e)), *δ* is almost constant while *γ*_1_, *γ*_2_, and *k* change slightly with increasing CWP. The radiative damping of the bright mode resonator, *γ*_1_, varies for CWP between C_2_ and C_5_. The non-radiative damping of the dark mode resonator, *γ*_2_, changes from 3.8 THz at C_0_ to 4.1 THz at C_5_. At the same time, *κ* decreases from 5.2 to 5 THz^2^ with increasing CWP. These variations in fitting parameters indicate that the physical properties of the analyte may affect the local field on the surface of the PGPP@MS biosensor, changing both the self- and mutual coupling of the bright and dark modes.

To characterize the sensing performance quantitatively, Δ*V*_T_ = *V*_T0_ − *V*_Ti_ (*V*_T0_ is transmission value at C_0_, *V*_Ti_ is transmission value at C_i_) and Δƒ_n_ = |*f*_ni_ − *f*_n0_| (*f*_ni_/*f*_n0_ is for frequency resonances with and without protein) were calculated between the bare sample and each CWP from C_1_ to C_5_ at frequencies *f*_1_–*f*_5_. Δ*V*_T_ is shown in Figure 4(a), and Δƒ is shown in Figure 4(b).

With increasing CWP, the value of Δ*V*_T_ at *ƒ*_1_ is almost unchanged, Δ*V*_T_ at the *ƒ*_2_ peak increases slightly, and Δ*V*_T_ at *ƒ*_3_–*ƒ*_5_ increases significantly. Δ*V*_T_ reaches a maximum value of 0.4 at *ƒ*_4_ for a CWP of 316 μg/mL (C_4_).

We selected the total transmission change at all frequency points, Δ*V*_T total_, for each CWP as the first biosensing index of the PGPP@MS platform. It is shown in Figure 4(d) (blue axis and markers). A significant change in Δ*V*_T total_ was observed with increasing CWP. When the concentration was 6.25 ng/mL (C_1_), Δ*V*_T total_ was significantly different from the 0 to at C_0_. Δ*V*_T total_ increases remarkably between concentrations C_1_ and C_2_ (2.52 μg/mL). When the concentration further increases to 316 μg/mL (C_4_), Δ*V*_T total_ reaches about 82%.

The peaks and minima of the transmission spectrum also change with CWP. At the low end of the wave band, terahertz waves have low energy and are insensitive to environmental changes. Even at high CWP, almost no shift of *ƒ*_1_, ƒ_2_, or *ƒ*_3_ takes place (Figure 4(b)). However, when the CWP is C_4_, Δ*ƒ*_4_ increases, then reaches a maximum value of 96 GHz at C_5_. Δ*ƒ*_5_ increases with increasing CWP to a maximum value of 224 GHz at C_4_, then decreases.

The total frequency shift Δ*ƒ*_total_ of all frequency points is shown in Figure 4(d) (purple axes and markers) as the second biosensing index of the PGPP@MS platform. Δ*ƒ*_total_ increases with increasing CWP, reaching 350 GHz at 316 μg/mL whey protein (C_4_). The result indicates that the PGPP@MS biosensor can detect frequency shifts as a useful sensing dimension.

The main sensing mechanism may be a change in the ultrasensitive photoconductivity properties of the MOSLS layer of the device based on the whey-protein-induced ED effect. This effect occurs when free electron/hole charges induced by electrostatic field excitations replace those normally supplied by a donor/acceptor dopant species. The distinct merit of ED is that the carrier concentration and polarity are tunable via external excitation.

Additionally, the phase change Δ*P* with and without whey protein was obtained for CWP ranging from C_1_ to C_5_, as shown in Figure 4(c). It is interesting to note that the dependence of phase difference on frequency is quasilinear. Consequently, the slope of the linear dependence can be considered a third biosensing index for PGPP@MS platforms. This is shown in Figure 4(d), where it can be seen that the change in slope is larger between lower concentrations. However, when the concentration grows from 46.8 μg/mL (C_3_) to 316 μg/mL (C_4_), the slope changes less rapidly. These results reveal that the phase is also altered after the introduction of protein, mainly because of the change in conductivity of perovskite. The maximum phase difference was 360° at 1.25 mg/mL (C_5_) with a slope of ∼400. Additionally, to verify this explanation, similar devices without perovskite were synthesized and the phase was measured under the same conditions. (See the Supplementary information for details.) The phase difference is almost zero as the CWP increases. Therefore, our claim that the phase change of the PGPP@MS biosensor is mainly caused by perovskite has been verified.

These results show that the PGPP@MS biosensor can function as a multidimensional biosensor capable of detecting Δ*V*_T_, Δ*ƒ* and Δ*P* in the presence of proteins.

### 3.3 Physical rationale for the sensing mechanism

To provide a physical rationale for the PGPP@MS device’s sensitivity, we propose a mechanism by which the ED effect [33] can be induced by protein molecules, illustrated in Figure 5. Without whey protein (Figure 5(a)), the energy band of perovskite is in its intrinsic state, and the initial Fermi level (*E*_F0_) of the p-type graphene is in the valence band (Figure 5(c) and (e)) [45, 46]. Usually, protein aggregates have a positive net charge because amino acids exist in the form of ions in aqueous solution [47, 48]. When a positively charged protein is dropped onto the upper graphene surface as an analyte, its electrostatic field will cause charge accumulation in the graphene and perovskite layer (see Figure 5(b)). Consequently, the Fermi level shifts from *E*_F0_ to *E*_F1_ (compare Figure 5(c) and (d)), and the conductivity of graphene gradually increases, resulting in lower transmission, as in Figure 4(d). The electric field increases with CWP, increasing the strength of the ED response.

Simultaneously, the protein-induced ED effect may also cause changes in the conductivity of the perovskite layer by changing its charge polarity and carrier concentration; or in other words, by changing its Fermi level (see Figure 5(f)).

To understand the physical mechanism of the frequency shifts, we used modified perturbation theory to analyze the relationship between the Δ*ƒ* values and changes in the dielectric environment. The relative change in the angular frequency Δ*ω* can be calculated by [11, 49], [50], [51]:

(3) $$\frac{\Delta \omega_{AM}}{\omega_{AM}}=-\frac{1}{2}\frac{\int_{0}^{h}E_{AM}\left(r\right)\cdot \left(\epsilon -1\right)\cdot E_{AM}\left(r\right)dr}{\int_{0}^{\infty }\left({\left|E_{AM}\left(r\right)\right|}^{2}\right)dr},$$

where *E*_AM_ is the electric field in the original metasurface, and *ε* is the equivalent dielectric constant of PGPP@MS. Because the electric field decays exponentially along the direction normal to the metamaterial, the frequency shift calculated from Eq. (3) scales with the amount of analyte as follows:

(4) $$\frac{\Delta \omega_{AM}}{\omega_{AM}}\propto \frac{\left(\epsilon -1\right)\cdot h}{l}=\Delta \epsilon \cdot \frac{h}{l}\propto \Delta \epsilon ,$$

where Δ*ε* is the difference in the equivalent dielectric constants with and without whey protein, h is the thickness of the monolayer of graphene (∼1 nm), and *L* is the penetration depth of the THz field. It can be concluded that the conductivity of graphene and perovskite changes because of the incorporation of protein, leading to an alteration of the dielectric environment of the metasurface and an increase in Δ*ƒ* with increasing CWP. Both the mobility and concentration of charge carriers in graphene will reach a maximum and saturate even if the CWP increases further, hence why Δ*ƒ*_5_ reached a maximum at C_4_, then decreased.

### 3.4 Light-induced reversible sensing

Until now, reuse of, or reversible sensing by, THz metasurfaces biosensors has been seldom reported. However, by tuning the conductivity of the MOSLS with a laser, the transmission spectrum can in principle be restored to the state without protein. Our work provides an opportunity to achieve reversible sensing in the THz region. Because of the poor thermal stability of whey protein, laser illumination may degrade it, reducing the net charge and weakening the ED effect on the MOSLS.

To demonstrate reversible sensing, we measured the transmission value (*V*_T_) of the biosensor at frequencies *f*_1_–*f*_5_ under illumination at wavelengths *λ* = 405 nm, 532 nm, and 808 nm and three different optical fluxes, *F*_op(1, 2, 3)_. Pentagonal radar maps of the change in *V*_T_ are plotted in Figure 6. *V*_T_ is shown in gray for the bare sensor (C_0_) and in red for the highest CWP tested, C_5_, because it showed a clear response at all five frequencies (see Figure 4(a)). (The corresponding transmission spectra are shown in Figures S3–S5.) *V*_T_ after optical exposure is shown in yellow for 405 nm, green for 532 nm, or purple for 808 nm. As *F*_op_ increases (across rows in Figure 6), the optically induced *V*_T_ gradually increases from the initial value marked in red (corresponding to C_5_) until it recovers to the value without protein, marked in gray.

At 405 nm and *F*_op(1)_ = 1.1 mW/cm^2^, only the *V*_T_ of *ƒ*_5_ increases. The main reason is that the laser power is too low to significantly influence the conductivity of the MOSLS. The *V*_T_ of all peaks increases for *F*_op(2)_ = 11.5 mW/cm^2^. At *F*_op(3)_ = 103.5 mW/cm^2^, the transmission values of all peaks return almost completely to their original values. The *V*_T_ values behave similarly at 532 nm and 808 nm, indicating that protein-induced transmission changes are indeed reversible.

It is known that the absorption wavelength of perovskite is less than 780 nm, so while it may respond to light at 405 nm and 532 nm, it should not respond to illumination at 808 nm. However, the device shows responses to all three wavelengths, implying that graphene may be the main contributor to the conductivity of the MOSLS under optical excitation.

Theoretically, the conductivity of graphene (*σ*_Gr_) is determined by the carrier densities, i.e., by the Fermi energy *µ*, and their effective temperature *T* [52]. Assuming that *σ*_Gr_ is a DC, low-signal-frequency conductivity determined by short-range scattering on defects and acoustic phonons, the scattering time *τ* = *τ*_0_ (*T*_0_/*рʋw*) ∝ 1/*p* [53], where *τ*_0_ is the characteristic short-range scattering time [54]. Then one can obtain the following formula:

(5) $$\sigma_{Gr}=\frac{2\sigma^{0}}{1+e^{-\frac{\mu }{T}}},$$

Where $\sigma^{0}=e^{2}T_{0}\tau_{0}/\pi h^{2}$ is the intrinsic conductivity under without any excitation conditions, *T* = *T*_0_ and *µ* = 0. At relatively weak irradiation, |*µ*| « *T*_0_, *T*, and Eq. (5) yields

(6) $$\sigma_{Gr}≅\sigma^{0}\left(1+\frac{\mu }{2T}\right).$$

Laser excitation can drive electrons from the photoexcited electron-hole pairs to accumulate in the graphene layer, leading to a shift of the Fermi level from *E*_F1_ (Figure 5(d)) in the valence band toward the Dirac point caused by a reduction in *μ*. Meanwhile, the carriers inevitably cause a temperature increase. From Eq. (6), the conductivity of graphene decreases with enhanced optical pumping, and the transmission values of all the frequency peaks decrease. In summary, *V*_T_ increases after laser excitation, permitting reversible sensing.

## 4 Conclusions

We have designed, fabricated, and characterized a novel THz biosensor. It comprises a metal oxide semiconductor-like structure based on patterned graphene–polyimide–perovskite integrated with an EIT-like metasurface. Our PGPP@MS biosensor successfully detected whey protein concentrations as low as 6.25 ng/mL by detecting changes of frequency, amplitude, and phase, demonstrating multidimensional biosensing. Furthermore, Δ*V*_T_, Δ*ƒ*, and Δ*P* increased significantly with whey protein concentration; Δ*V*_T total_, Δ*ƒ*_total_, and Δ*P* approached 82%, 400 GHz, and 360° at 316 μg/mL whey protein, respectively. To explain the internal mechanism of ultra-sensitive, multidimensional biosensor performance, we carried out a theoretical analysis of the changes of the sensor’s optoelectronic properties based on the ED effect. In addition, we have successfully realized reversible biosensing using lasers with several different wavelengths. This work could be of great importance for applications of THz metasurfaces in the field of biosensing.

## Supplementary Material

Supplementary Material
