# Supplementary material for: Ultrasensitive, light-induced reversible multidimensional biosensing using THz metasurfaces hybridized with patterned graphene and perovskite
Source: Nanophotonics. 2022 Feb 11;11(6):1219–30. doi: 10.1515/nanoph-2021-0816 (PMC11501886; doi:10.1515/nanoph-2021-0816)
Supplement: Supplementary file 1 — Supplementary Material [file j_nanoph-2021-0816_suppl.docx]

**Ultra-sensitive, light-induced reversible multidimensional biosensing using THz metasurfaces hybridized with patterned graphene and perovskite**

Haiyun Yao^a^†, Zhaoqing Sun^b^† Xin Yan^c^*, Maosheng Yang^d^*, Lanju Liang^a^*, Guohong Ma^e^, Ju Gao^a^, Tenten Li^f^, Xiaoxian Song^g^, Haiting Zhang^g^, Qili Yang^a^ , Xiaofei Hu^a^, Ziqun Wang^a^, Zhenhua Li^a^ and Jianquan Yao^f^

*^a^ School of Opto-electronic Engineering, Zao zhuang University, Zao zhuang 277160, China*

*^b^Faculty of Materials and Manufacturing, Beijing University of Technology, Beijing, 100124, China.*

*^c^School of Information Science and Engineering, Zao zhuang University, Zao zhuang 277160, China*

*^d^School of Electrical and Optoelectronic Engineering, West Anhui University, Lu'an 237000, China*

*^e^ Department of Physics, Instrumental Analysis & Research Center, Shanghai University, Shanghai 200444, China*

*^f^* *College of Precision Instruments and Opto-electronics Engineering, Tianjin University, Tianjin 300072, China*

*^g^ Institute of Micro-nano Optoelectronics and Terahertz Technology, Jiangsu University, Zhenjiang, 212013, China*

**Ⅰ. Materials and apparatus**

Three layers graphene grown by CVD method was bought from Vigon Technology (Hefei, China). whey protein was purchased from Aladdin Biochemical Technology (Shanghai, China). Metal halide perovskite (MAPbI_3_) was synthesized according to the procedure previously reported[1]. Equimolar mixtures of the as-synthesized MAI and PbI2 in γ-butyrolactone (99+%, Acros Organics) were stirred overnight at RT/60 °C to obtain a 20 wt % solution.

The Raman measurements were carried out by a LabRAM HR Evolution Raman microscope system (Horiba Jobin Yvon, Piscataway, USA), in which a He–Ne laser with a wavelength of 514 nm was utilized as the excitation source. XRD characterization was performed with a Thermo X’tra powder diffractometer equipped with Cu Kα radiation and a Thermo Electron Si (Li) solid state detector. Diffraction pattern were collected by 0.05° 2θ steps and counting times ranging from 2 to 10 s per step. In order to measure the XRD spectra of the perovskite powders, obtained films were carefully scratched from their substrate by an edged glass slide

Fig. S1 and S2. shown an 8f confocal terahertz time-domain spectroscopy system (THz-TDs) was used in the measurements. In the measurements, the normally incident THz wave is along +z. We introduced dry air into the THz-TD_S_ setup to maintain a constant environment. The room temperature is about 24℃; the room humidity is about 2.7%. For the THz-TD_S_ setup, LED-optically pumped all-fiber femtosecond laser is used to generate terahertz wave time-domain spectroscopy. Bandwidth is 0.2THz-2THz, and SNR is about 60 dB. The laser centered at 1560 nm wavelength with 100 fs pulse duration and 100 MHz repetition rate. As shown in Fig. S1, the laser (with 100 mW power) is split into equal intensity beams. One beam excites a biased photoconductive antenna to generate the THz transient, while the other services as a gate in a photoconductive antenna to detect the THz pulse. The THz time scan of 40 ps was used to obtain a spectral resolution of 40 GHz. For the external stimuli system, a 532 nm laser (green light) services as an external optical pump. A probe system was applied voltage to graphene, as shown in Fig. S2.


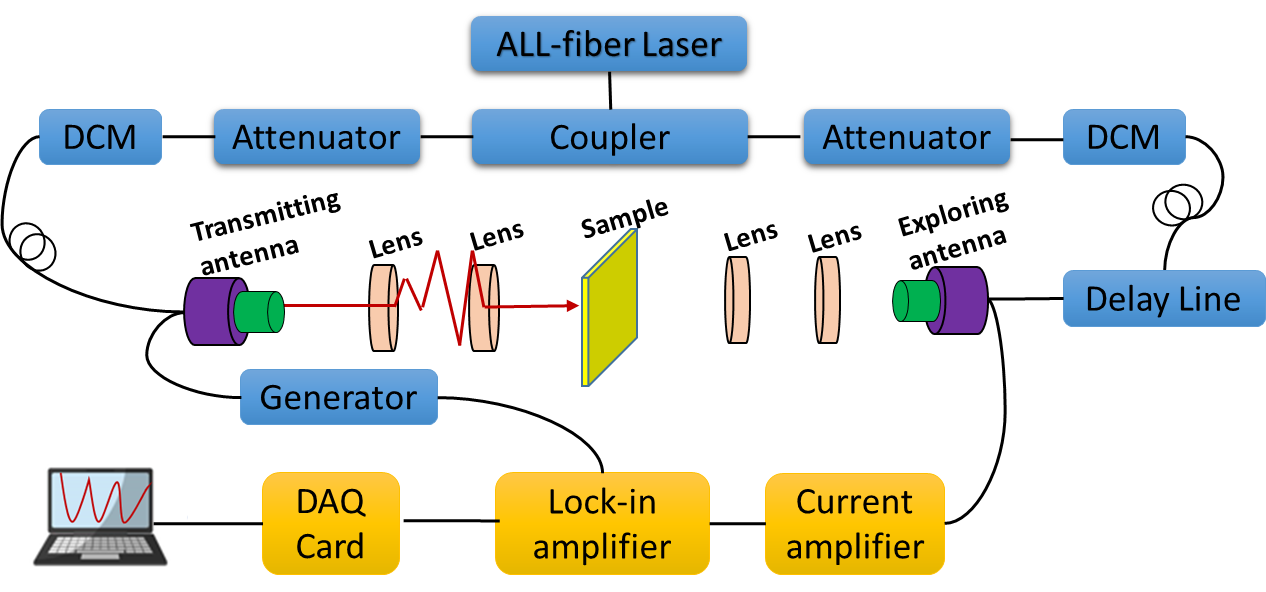


**Figure S1** The schematic view of all-fiber THz-TDS measurement setup


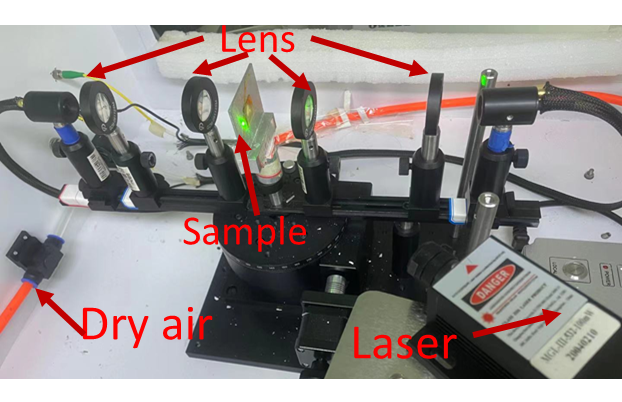


**Figure S2** Experimental photoelectric equipment: THz-TDs system

**Ⅱ. The PGP@MS sample**

To verify our explanation, the device based on patterned graphene-polyimide-metasurfaces(PGPP@MS) was synthesized. The phase difference(ΔP) between the sample with and without whey protein, for CWP ranging from 63.8 ng/ml to 1.42 mg /ml, can be measured as shown in Fig.S3. It is interesting to note that the dependence of ΔP on frequency is quasi-linear. However, the ΔP corresponding to different concentrations almost unchange, and these results are very different from the results of sample mentioned in the main text. Therefore, it can be proved that the phase modulation achieved in the PGPP@MS samples mentioned in the main text is mainly based on perovskite.





**Figure S3** Phase difference between the PGPP@MS sample with and without whey protein under whey protein concentration ranging from 63.8 ng to 1.42 mg/ml

**III. The PGP@MS sample**


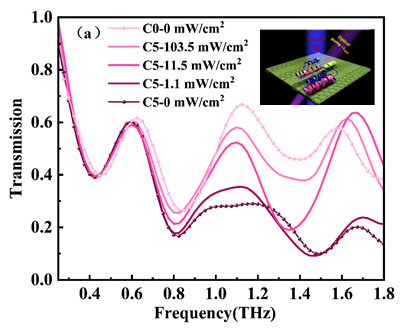

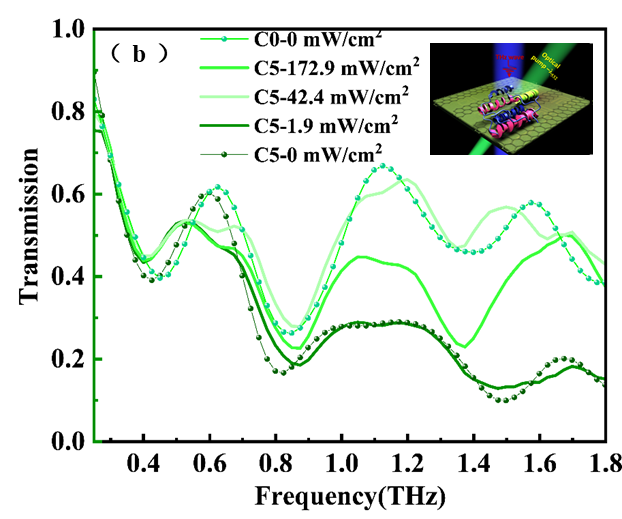


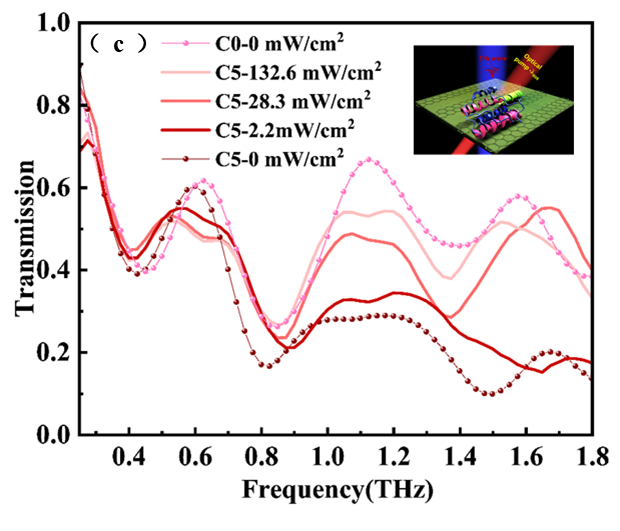


**Figure S4** Experimentally measured THz transmission spectra of PGPP@MS biosensor with whey protein concentrations is1.25mg/ml and bare under different optical flux (Fop) with different wavelength laser：(a) λ=405nm ;(b) for λ=532 nm; (c) for λ=808 nm; insert: Schematic diagram of the PGPP@MS biosensor under THz beams and external excitation optical pump.

**References**

[1] C. Li, W. Li, S. Duan, J. Wu, B. Chen, S. Yang, R. Su, C. Jiang, C. Zhang, B.J.A.P.L. Jin, Electrically tunable electromagnetically induced transparency in superconducting terahertz metamaterials, 119(5) (2021) 052602.
